# Do we need a re-TUR after en bloc resection of T1 stage bladder cancer?

**DOI:** 10.1007/s00345-024-05175-y

**Published:** 2024-08-08

**Authors:** Stephan Levy, Sarah Péricart, Anne Sophie Bajeot, Sami Fakhfakh, Marine Lesourd, Michel Soulié, Géraldine Pignot, Mathieu Roumiguié

**Affiliations:** 1 Department of Urology, CHU-Institut Universitaire du Cancer de Toulouse Oncopole, 1 Av Jean Poulhès, 31059 Toulouse, France; 2 Department of Pathology, CHU-Institut Universitaire du Cancer de Toulouse, Toulouse, France; 3 Department of Surgical Oncology 2, Institut Paoli-Calmettes, Marseille, France; 4 Department of Urology, Clinique La Croix du Sud, Quint Fonsegrives, France

**Keywords:** Urology, Oncology, Bladder tumor, En bloc resection, High-grade T1, Second look, Trans-urethral bladder resection

## Abstract

**Background:**

A second look trans-urethral resection of the bladder (re-TUR) is recommended after the diagnosis of T1 high grade (T1HG) bladder cancer. Few studies have evaluated the results of re-TUR after a first en bloc resection (EBR) and none of them have specifically reported the pathological results on the field of previous T1 disease.

**Objective:**

To report the rate of upstaging and the rate of residual disease (RD) on the field of T1HG lesions resected with EBR.

**Materials and methods:**

Between 01/2014 and 06/2022, patients from 2 centers who had a re-TUR after an EBR for T1HG urothelial carcinoma were retrospectively included. Primary endpoint was the rate of RD including the rate of upstaging to T2 disease on the scar of the primary resection. Secondary endpoints were the rate of any residual disease outside the field.

**Results:**

Seventy-five patients were included. No muscle invasive bladder cancer lesions were found after re-TUR. Among the 16 patients who had a RD, 4 were on the resection scar. All of these lesions were papillary and high grade. RD outside the field of the first EBR was observed in 12 patients.

**Conclusion:**

After EBR of T1HG disease, none of our patients had an upstaging to MIBC. However, the rate of RD either on and outside the field of the EBR remains quite significant. We suggested that predictive factors of residual papillary disease (number of tumors at the initial TUR and concomitant CIS) might be suitable to select patient who will benefit of the re-TUR.

## Introduction

Trans-urethral resection of the bladder (TUR) is considered the cornerstone of the management of non-muscle invasive bladder cancer (NMIBC) [1]. In case of T1 disease with presence of muscle, the guidelines recommend to perform a second look resection (re-TUR) of the primary tumor scar to decrease the risk of missing an upstaging to a muscle invasive bladder cancer (MIBC). The rational of re-TUR is based on large retrospective studies and meta-analysis which reported 30% rate of muscle-invasive disease after a T1 tumor resected with conventional TUR (cTUR) [2–4].

En bloc resection (EBR) is attracting increasing interest as an alternative to cTUR for the following reasons: (1) its non-inferiority in terms of oncological outcomes (2) its lower risk of perioperative complications (3) EBR provides an intact tumor specimen with a respect of the micro-architecture of the bladder layers. That advantage allows the pathologist a better quality of muscle analysis such as a substaging T1a and T1b of T1 [5] (4) tumor removal via EBR resulted in a higher rate of detrusor muscle (DM) in the pathologic specimen [6].

Thus, we assumed a low risk of MIBC upstaging at re-TUR after T1 disease EBR and we hypothesized that re-TUR may not benefit from all patients. Indeed, re-TUR is associated with an expected risk of complication from the second anesthesia and procedure [7]. In fact, morbidity and mortality are higher in patients aged over 80, patients frequently found in the bladder cancer population [8].

The main objective of this retrospective observational study was to report the rate of upstaging and the rate of residual disease (RD) on the field of T1 disease diagnosed with EBR technique.

## Materials and methods

### Study design and endpoints

In this retrospective observational multicentric study (Toulouse University Hospital and Institut Paoli-Calmettes Marseille), we selected patients who underwent re-TUR for T1 urothelial carcinoma initially resected with EBR between January 2014 and June 2022. Patient could have multiple T1 disease or concomitant papillary Ta and CIS lesions. Only patients with a complete EBR (no macroscopic RD and presence of muscle) and with conventional urothelial carcinoma were included. Data was collected on clinical variables, tumor characteristics and operative data at the EBR and re-TUR. We carefully distinguished from pathological report of the restating TUR whether the location of the residual tumor was on or outside the field of the first tumor.

### Objectives

The primary endpoint was the rate of RD including the rate of upstaging to muscle invasive bladder cancer on the scar of the primary resection at the re-TUR.

Secondary endpoints included the rate of any papillary residual tumor on the scar and in other location of the bladder at the re-TUR.

### Statistical analysis

Continuous variables were expressed as median or mean (± standard deviation) while categorical variables were presented as absolute number and percentage. Mann–Whitney *U*-test and chi-squared tests were used to compare quantitative and qualitative variables. Kaplan–Meier analysis with log-rank test and Cox regression were performed for recurrence and progression survival. Recurrence was defined as a new histologically proven urothelial lesion while the progression was defined as muscle invasive bladder cancer. The presence of Carcinoma in Situ (CIS) alone in the re-TUR was not considered as a RD because we assumed that CIS couldn’t be completely resected in the initial TUR. The time to recurrence and progression was defined as the time from re-TUR to the event. Follow-up time was defined as the time from re-TUR to the date of the last visit. We performed univariate and multivariate logistic regression (including variables that reached *p* < 0.1 in univariate analysis) to assess risk factors for recurrence and progression. Statistical analyses were performed using R studio. All tests were two-sided with a significance level at *p* < 0.05. Ethics approval was acquired (study registration number RnIPH 2022–54).

## Results

### Population characteristics

**Fig. 1:**
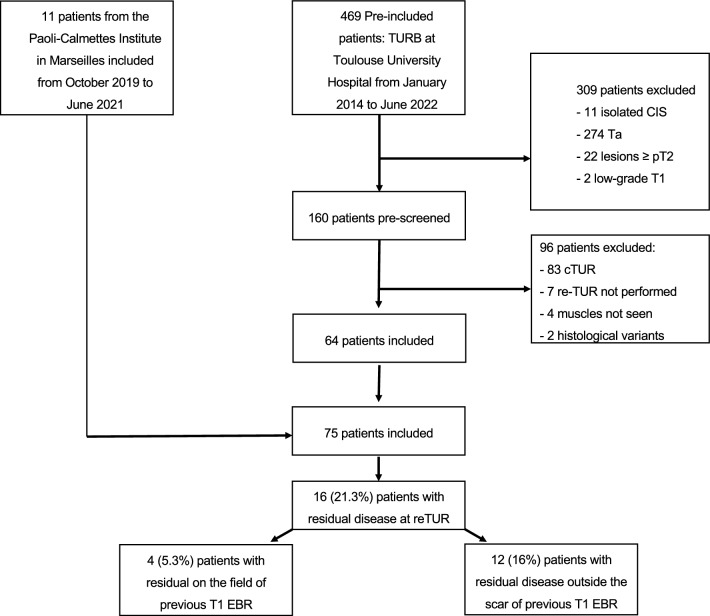
Flow-chart depecting the selection (inclusion and exclusion criteria) of patients. TUR: trans-urethral bladder resection; cTUR: conventional TUR; CIS: carcinoma in situ

In overall cohort, 75 were included (Fig. 1). Enhanced cystoscopy technique was used in 58 (77%) patients. At the first EBR, 40 (53.3%) patients presented multiple papillary tumors and 27 (36%) patients had more than one T1 lesion. Tumor size was larger than 3 cm in 49 (65.3%) patients and concomitant CIS was diagnosed in 22.4% (17) of patients. Re-TUR was performed at 7.6 (± 2.9) weeks after the EBR. The mean follow-up duration of the overall population was 22.4 (±17.3) months (Table 1).

**Table 1:** Overall population characteristics stratified by the presence/absence of papillary (Ta/T1) residual disease at re TUR

	Overall cohort	Papillary residual disease		*p*
		No	Yes	
	*n* = 75	*n* = 59	*n* = 16	
Age (years, mean (SD))	71.5 (9.7)	70.0 (9.82)	77.1 (7.10)	0.009
Age > 70 years (*n*, %)	40 (53.3)	27 (67.5)	13 (32.5)	0.025
Sex, *n* (%)				
Male	67 (89.4)	52 (77.6)	15 (22.4)	0.850
Female	8 (10.6)	7 (87.5)	1 (12.5)	
Enhanced cystoscopy (*n*, %)				
White light	17 (22.7)	13 (76.5)	4 (23.5)	1.000
Blue light/NBI	58 (77.3)	46 (79.3)	12 (20.7)	
Number of tumour (mean (SD))	1.68 (1.22)	1.44 (1.07)	2.56 (1.36)	0.001
Tumor size (cm), *n* (%)				
<3 cm	26 (34.7)	21 (80.8)	5 (19.2)	0.978
≥3 cm	49 (65.3)	38 (77.6)	11 (22.4)	
Concomitant CIS (*n*, %)	17 (22.7)	10 (58.8)	7 (41.2)	0.053
Detrusor muscle presence (*n*, %)	75 (100)	59 (100)	16 (100)	1
Time before re TUR (weeks, mean (SD))	7.63 (2.9)	7.41 (2.47)	9.35 (5.5)	0.025
Follow-up duration (month, mean (SD))	22.4 (17.3)	21.25 (16.94)	26.44 (18.30)	0.288

Analysis of factors associated with to the presence of residual disease re-TUR

*TUR* trans-urethral bladder resection, *NBI* narrow band imaging, *T1HG* high grade T1 lesion, *CIS* carcinoma in situ

#### Rates of upstaging and residual disease on the field of the EBR

**Fig. 2:**
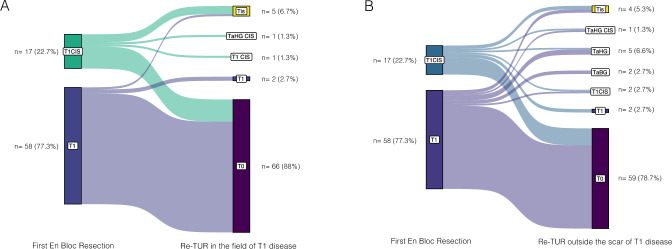
Sankey diagrams of pathologic result of the re-TUR in the field of the primary T1 (**A**) and of pathologic result of the re-TUR outside the scar (**B**). CIS: carcinoma in situ; HG: high grade; LG: low grade

At the re-TUR, 4 (5.3%) patients had a RD in the field of the T1 disease EBR. Three (4%) of them had a T1 RD while one (1.3%) had Ta RD (Fig. 2A). At second TUR, the DM was present in 92.0% (69/75) of the specimen, and we did not observe any upstaging disease to MIBC on the field of the primary T1 tumor.

#### Rates of upstaging and residual disease outside the field of the primary T1

RD was found in 12 (16%) patients outside the location of the first EBR. No MIBC was diagnosed. Four (5.3%) patients had T1 RD and 8 (10.7%) had Ta RD (Fig. 2B). The DM was present in 93.3% (70/75) of specimens.

##### Any residual disease in the bladder impacted the recurrence free survival (RFS)

**Fig. 3:**
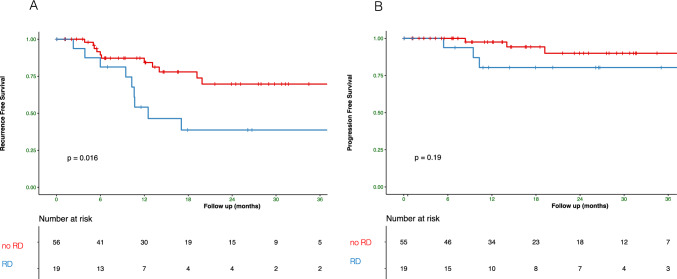
Kaplan–Meier curves for recurrence-free (**A**) and progression-free (**B**) survival of patients with (blue) and without (red) any residual disease at re-TUR

We then focused our analysis on whether the presence of any residual papillary (*n* = 16, 21.3%) (Ta/T1) disease at re-TUR was associated with worse oncologic outcomes. Patients with RD at re-TUR experienced more recurrences than the other group without RD (9/16 (56.2%) vs. 11/59 (18.6%), *p* = 0.007) (Table 1). We found that RFS of patients with RD was shorter than those of the group without RD (*p* = 0.016; Fig. 3A) while no difference was observed in PFS (Fig. 3B).

##### Risk factor of residual disease

The presence of RD was associated with an increasing time between the first and second TUR (no RD = 7.41 weeks vs. RD = 9.35; *p* = 0.025). Patient with RD were older (77.1 vs. 70.0; *p* = 0.009) with a greater number of tumors (2.6 vs. 1.4; *p* = 0.001) than those without RD. Concomitant CIS was more likely associated with RD without reaching the significant level (43.8% vs. 16.9%; *p* = 0.053) (Table 1). We included these 4 factors (age, number of tumors, concomitant CIS and Time to re-TUR) in a multivariable analysis and we observed that the presence of CIS (Odds Ratio [OR] 4.4 [95% CI 1.2–16.9]; *p* = 0.025) and the number of tumors (OR 2.2 [95% CI 1.4–3.8]; *p* = 0.001) remained independent predictive factors of RD (Table 2).

**Table 2:** Multivariable logistic regression analyses for prediction of residual disease at re TUR; 95%

Variables	Multivariable		
	OR	95% CI	*p*
Age > 70 years	3.23	0.9–13.9	0.09
Concomitant CIS	4.4	1.2–16.9	0.025
Number of tumour	2.2	1.4–3.8	0.001
Time before re TUR	0.98	0.80–1.20	0.86

*OR* odds ratio, *CI* 95% confidence interval, *CIS* carcinoma in situ

## Discussion

In this study, we reported the rate of RD at the re-TUR after EBR of T1 disease, separately in the field and elsewhere in the bladder. Using this method, we could estimate the rate of upstaging of T1 disease.

Regarding the pathological result on the field of EBR T1 disease, we did not observe any upstaging to T2 disease while we found 4 (5.3%) patients with RD including 3 patients with T1 stage disease. Our results are consistent with the results of EBR technic recently published. A systematic review including 539 patients reported a rate of upstaging after EBR at 0.0% (95% CI 0.0–0.5%) [9]. Only one study reported the RD location, 6 out of 50 patients had residual tumor at the original site of T1 disease [10]. This result support that initial EBR assigns accurately the pathological stage as compare to cTUR [11]. A recent phase 3 trial also revealed a lower rate of residual papillary (Ta/T1) disease in the EBR arm [6]. These results are also in line with those established by the recently published phase 3 trial showing that EBR could induce a reduction in the 1-year recurrence rate of NMIBC [12].

We observed a significant (29%) rate of RD elsewhere in the bladder while the initial resection was deemed complete. After cTUR for T1 disease, the rate of RD ranged from 20 to 71% and was reported at 24% when the first TUR was deemed complete [13]. With the EBR, the residual tumor rate varied from 0% to 29.3% [9]. As previously reported in the literature, we observed that the risk of residual tumor was increasing with the number of tumor [14] and concomitant CIS [15].

Our study showed a very low risk of RD in the field whereas we observed a significant risk of RD elsewhere in the bladder.

Then, we evaluated if the overall residual papillary (Ta/T1) disease of the bladder may impact the oncologic outcomes. We observed, as reported by Soria et al., an association between the presence of RD and the RFS [15] which is so called a prognosis factor.

We therefore proposed to classify the risk of RD into 3 different groups. The first group would be composed of patients with no risk factors of residual papillary disease (unique T1 without CIS). Among the 30 patients (40%), none of them had RD. Conversely, patients with both risk factors (multiple lesions and concomitant CIS) would be defined as a high-risk group. This group represents 12 patients (16%) in our cohort: 5 of them (41.6%) had RD. Finally, considering an intermediate group of 33 patients (44%) with only one out of the 2 risk factors.

As the risk of RD is very low in patients without risk factors, these patients might be good candidates for replacing routine second TUR with non-invasive methods such as ambulatory cystoscopy, cytology and/or biomarkers [16]. Implementation of the checklist and use of enhanced cystoscopy were known to improve the quality of both the detection and the resection [17, 18].

In this perspective, we could design a trial with 2 arms: the experimental arm with cystoscopy and urinary cytology versus a control arm with systematic second look resection before endo-vesical BCG instillations.

This study is not devoid of limitations as the retrospective design and the small number of patients included. Regarding risk factors of RD, multivariate analysis results should be taken with caution regarding the small number of events (*n* = 16). Moreover, surgeon expertise and selection criteria for EBR technique weren’t available in the retrospective data collection which can lead to a selection bias. However, we carefully selected only T1 disease and interpreted the pathological result according to the site of the resection. We considered this point as a strength of our study allowing to distinguish the clinical information sending by the re-TUR: Is the first TUR was incomplete? Is an aggressive T1 with residual disease on the field might be considered for more aggressive treatment such as radical cystectomy? Likewise, we did not consider as significant RD the presence of isolated CIS on the re-TUR because that will not change the indication of BCG instillations.

## Conclusion

We observed that none of our patients had MIBC on the re-TUR after EBR. The risk of residual tumor remains significant and has a significant impact on recurrence-free survival, suggesting the importance of continuing to achieve a second endoscopy in this indication. We found predictive factors of residual papillary disease on this re-TUR which could help select patients who will benefit more from second look resection. These factors need to be validated in further studies.
